# Interobserver variation in the interpretation of magnetic resonance enterography in Crohn’s disease

**DOI:** 10.1259/bjr.20210995

**Published:** 2022-05-12

**Authors:** Gauraang Bhatnagar, Sue Mallett, Laura Quinn, Richard Beable, Helen Bungay, Margaret Betts, Rebecca Greenhalgh, Arun Gupta, Anthony Higginson, Rachel Hyland, Rajapandian Ilangovan, Hannah Lambie, Evgenia Mainta, Uday Patel, James Pilcher, Andrew Plumb, François Porté, Harbir Sidhu, Andrew Slater, Damian Tolan, Ian Zealley, Steve Halligan, Stuart Taylor

**Affiliations:** Centre for Medical Imaging, Charles Bell House, University College London, London, UK; Centre for Medical Imaging, Charles Bell House, University College London, London, UK; Institute of Applied Health Research, NIHR Birmingham Biomedical Research Centre, College of Medical and Dental Sciences, University of Birmingham, Birmingham, UK; Department of Radiology, Portsmouth Hospitals NHS Trust, Portsmouth, UK; Department of Radiology, Oxford University Hospitals NHS Trust, Oxford, UK; Department of Radiology, Oxford University Hospitals NHS Trust, Oxford, UK; Department of Radiology, St George’s University Hospitals NHS Trust, London, UK; Intestinal Imaging Centre, St Mark’s Hospital, LNWUH NHS Trust, Harrow, UK; Department of Radiology, Portsmouth Hospitals NHS Trust, Portsmouth, UK; Department of Radiology, St James’s University Hospital, Leeds Teaching Hospitals NHS Trust, Leeds, UK; Intestinal Imaging Centre, St Mark’s Hospital, LNWUH NHS Trust, Harrow, UK; Department of Radiology, St James’s University Hospital, Leeds Teaching Hospitals NHS Trust, Leeds, UK; Intestinal Imaging Centre, St Mark’s Hospital, LNWUH NHS Trust, Harrow, UK; Intestinal Imaging Centre, St Mark’s Hospital, LNWUH NHS Trust, Harrow, UK; Department of Radiology, St George’s University Hospitals NHS Trust, London, UK; Centre for Medical Imaging, Charles Bell House, University College London, London, UK; Intestinal Imaging Centre, St Mark’s Hospital, LNWUH NHS Trust, Harrow, UK; Centre for Medical Imaging, Charles Bell House, University College London, London, UK; Department of Radiology, Oxford University Hospitals NHS Trust, Oxford, UK; Department of Radiology, St James’s University Hospital, Leeds Teaching Hospitals NHS Trust, Leeds, UK; Department of Radiology, Ninewells Hospital, Dundee, UK; Centre for Medical Imaging, Charles Bell House, University College London, London, UK; Centre for Medical Imaging, Charles Bell House, University College London, London, UK

## Abstract

**Objectives:**

To evaluate interobserver variability for diagnosis of disease presence and extent of small bowel and colonic Crohn’s disease using MR enterography (MRE)

**Methods:**

Data from the first 73 consecutive patients (mean age 32, 33F, 28 new diagnosis, 45 suspected relapse) recruited to a multicentre, prospective diagnostic accuracy trial evaluating MRE for small bowel Crohn’s disease were each read independently by three (from a pool of 20) radiologists. Radiologists documented presence and segmental location of small bowel Crohn’s disease and recorded morphological mural/extramural parameters for involved segments. Per patient percentage agreement for disease presence and extent were calculated against an outcome-based construct reference standard (averaged between pairs of readers). Prevalence-adjusted bias-adjusted κ (PABAK) was calculated.

**Results:**

Agreement for small bowel disease presence for new diagnosis/relapsed patients was 68%(κ = 0.36)/ 78% (κ = 0.56) and 43%(κ = 0.14)/ 53% for disease extent (κ = 0.07), respectively. For disease presence, all three radiologists agreed correctly with the reference standard in 41/59 (69%) of patients with small bowel involvement, and in 8/14 (57%) cases of without small bowel disease. Agreement was highest for multisegment disease, greater than 5 cm in length, with mural thickness>6 mm, and increased mural T2 signal. Agreement for colonic disease presence was 61% (κ = 0.21 fair agreement) for new diagnosis/ 60% (κ = 0.20, slight agreement) for relapsed patients.

**Conclusion:**

There is a reasonable agreement between radiologists for small bowel disease presence using MRE for newly diagnosed Crohn’s disease, and patients with suspected relapse, respectively. Agreement is lower for disease extent.

**Advances in knowledge:**

There is reasonable agreement between radiologists for small bowel disease presence using MRE for newly diagnosed (68%) Crohn’s disease, and patients with suspected relapse (78%). Agreement is lower for disease extent (43% new diagnosis and 53% suspected relapse).

## Introduction

Magnetic Resonance Enterography (MRE) has high diagnostic accuracy for Crohn’s disease presence and activity.^1–3^ Good technical quality is an important perquisite but, thereafter, accurate image interpretation is paramount. This relies upon perception of various stigmata of Crohn’s disease, including mural and peri-mural changes and extramural-complications.^4^ Many of these findings are subtle, especially in early, superficial disease, and therefore likely subject to most interpretative variation between radiologists. To date research on interobserver variability in MRE has mainly focused on agreement for various individual signs of Crohn’s disease and activity scores^5–7^ with only a few studies investigating interobserver variability for overall disease presence and activity on a per patient and per segment basis.^5,8,9^ Low interobserver agreement for disease presence and activity would be problematic as MRE is already widely disseminated in clinical practice.

The purpose of this study was to evaluate interobserver agreement for small bowel and colonic disease presence and activity using MRE datasets acquired as part of a prospective multicentre trial evaluating the diagnostic accuracy of MRE and SBUS in Crohn’s disease^2,10^

## Methods

### Study population

The METRIC (MR Enterography or ulTRasound In Crohn's disease) Trial was a multicentre, prospective cohort trial comparing the diagnostic accuracy of MRE and SBUS for the presence, extent and activity of enteric Crohn’s disease.^2,11^ The trial recruited two patient cohorts: (1) newly diagnosed and (2) established disease, clinically suspected of luminal relapse. Full ethical permission was obtained (NRES Committee South Central-Hampshire B, 13/09/2013, REC ref: 130054) and patients gave written consent.

### Study design

#### MRE protocol

MRE was performed as per usual clinical practice at individual sites and included a minimum dataset of sequences (Supplementary Material 1 - Appendix 1).

#### Radiologists

Twenty radiologists from seven of the eight recruitment sites participated in the current substudy. All were members of the British Society of Gastrointestinal and Abdominal Radiology (BSGAR), and held the Fellowship of the Royal College of Radiologists, with a minimum 1 year subspecialty training in gastrointestinal radiology.

### MRE dataset selection and interpretation

All MRE datasets acquired as part of the trial were uploaded to an online viewing platform (Biotronics 3Dnet, Biotronics 3D, London, UK), with functionality of a standard PACS system.

MRE datasets from the first 75 consecutive recruits were selected for the current substudy; two patient datasets were subsequently withdrawn as not having Crohn’s disease. As this study was a substudy of an ongoing larger trial, the number of patients was a pragmatic choice according to the available resources, and no power calculation was undertaken. Datasets were randomised by the trial statistician, ordered for interpretation and subsequently allocated to the radiologists. The trial statistician also ensured that no radiologist was allocated an examination they had interpreted for the main trial. Each dataset was read three times in total – read 1: by a local radiologist at the recruitment site (*i.e.,* the main trial read), and read 2 and 3: by two other radiologists from another site using the online viewing platform. The radiologists were blinded to all clinical information other than the patient cohort (new diagnosis or relapse).

All radiologists were asked to record segmental disease presence, activity, and extraenteric manifestations on a trial CRF (Supplementary Material 1- Appendix 2), based on their usual reporting practice. The small bowel and colon were divided into 4 and 6 segments, respectively (duodenum, jejunum, ileum, terminal ileum, rectum, sigmoid, descending colon, transverse, ascending and caecum) using previously published definitions.^12^ Radiologists documented their diagnostic confidence for disease presence in each bowel segment from 1 (least confident) to 6 (most confident). More detailed observations were collected for segments attracting a confidence score of 3 or more for disease presence. A laminated chart containing definitions and example images for all recorded observations was provided for reference during MRE interpretation (Supplementary Material 1- Appendix 2).

### Reference standard

The reference standard for disease presence and extent for each patient was that used by the main trial, i.e. an outcome-based, construct reference standard (Supplementary Material 1- Appendix 3).^2,10^

### Statistical analysis

The six-point confidence scale for disease presence and activity were converted to a binary outcome; “no disease/ not active” (confidence levels 1 and 2) or “disease present/ disease active” (confidence levels 3 to 6), mirroring the main trial analysis.^2^ Equivocal findings on MRE often result in similar clinical outcomes as positive findings: a trial of treatment or further investigation.

Datasets were grouped as positive or negative for disease presence according to the consensus reference, and interobserver agreement for disease presence and extent expressed as percentage agreement on a per patient level, averaged across pairs of readers. For disease extent, radiologists had to agree both on disease presence and (all) segmental location(s). Prevalence adjusted bias adjusted κ (PABAK) was calculated. The analysis was repeated for the subgroups of newly diagnosed and suspected relapse, and separately for small bowel and colon. κ statistics can be categorised as follows: 0.01–0.20, slight agreement; 0.21–0.40, fair agreement; 0.41–0.60, moderate agreement; 0.61–0.80, substantial agreement; and 0.81–0.99, almost perfect agreement.^13^ Segmental agreement was displayed graphically. Descriptive statistics for agreement between radiologists irrespective of concordance with the reference standard are also presented.

An exploratory analysis identified features associated with agreement between radiologists for small bowel disease. Specifically, all datasets were divided into those where all three radiologists agreed on disease presence/absence (irrespective of concordance with the reference standard), and those where only two radiologists agreed. The proportion of datasets in each agreement group was compared according to various patient (*e.g.,* Montreal classification) and imaging (*e.g.,* wall thickness, mural T2 signal) characteristics, and differences compared using univariable analysis.

A small proportion of the results have been published previously.^10^ The current report presents a more detailed description of study findings, necessarily set in context with a small proportion of results previously reported in Health Technology Assessment.^10^

## Results

### Demographic data

Two patients were withdrawn as the consensus reference standard meeting concluded they did not have Crohn’s disease, leaving 73 MRE datasets (28 and 45 new-diagnosed and relapsed patients, respectively) (Table 1). There were 219 individual interpretations (each of the 73 datasets read three times).

**Table 1.** Characteristics of patients according to patient cohort

Characteristics	All patients *N* = 73	
	New patients [n (%)] *N* = 28	Relapse patients [n (%)] *N* = 45
**Age – yrs., median (IQR**)	32 (24 to 48)	33 (23 to 46)
**Male**	16 (57)	24 (53)
**Previous enteric surgery**		
Yes	1 (4)a	27 (60)
**Colonoscopy available to consensus reference panel**	27 (96)	17 (38)
**Disease presence**		
Small bowel	26 (93)	33 (73)
Colon	14 (50)	17 (38)
**Disease duration**		
<1 year	NA	1 (2)
1–5 years	NA	12 (27)
6–10 years	NA	11 (24)
>10 years	NA	21 (47)
**Previous disease location** (**Montreal classification**)		
L1	NA	14 (31)
L2	NA	8 (8)
L3	NA	22 (49)
L4	NA	1 (2)
**Previous disease behaviour** (**Montreal classification**)		
B1	NA	24 (54)
B1p	NA	1 (2)
B2	NA	14 (31)
B3	NA	5 (11)
B3p	NA	1 (2)
**Inclusion criteria for relapse cohort patients**		
Raised CRP>8replace_with( >^−1^)	NA	21 (47)
Raised calprotectin>100	NA	3 (7)
Obstructive symptoms	NA	24 (53)
Abnormal endoscopy	NA	3 (7)

Of the 75 patients recruited for the interobserver study, 2 patients were withdrawn due not having Crohn’s disease at consensus stage.

NA – not applicable as characteristics are only relevant to relapse patients.

a Surgical resection for inflammatory mass 1 year prior to Crohn’s disease diagnosis.

### Radiologist characteristics and disease detection performance

The 20 participating radiologists had a median 10 years experience of specialist GI radiology practice, (interquartile range 6 to 11 years) (Table 2). 16 had more than 5 years experience (classified as experienced (E)), performing 167 reads, and four less than 5 years experience (classified as less experienced (LE)), performing 52 reads. In total, 180 of 219 reads (82%) agreed with the consensus reference standard for small bowel disease presence (136/167 (81%) for experienced radiologists and 44/52 (85%) for less experienced radiologists) and 143 of the 219 reads (65%) for small bowel disease extent (108/167 (65%) for experienced radiologists and 35/52 (67%) for less experienced).

**Table 2.** Radiologist experience in MRE

Radiologist	Recruitment site	Experience (Experienced (E) > 5 years, Less experienced (LE)<5 years)	Overall experience of MRE Imaging (at start of trial) [years]
1	1	E	13
2	2	E	11
3	2	LE	3
4	2	E	12
5	2	E	8
6	3	E	12
7	3	E	6
8	3	E	10
9	4	E	6
10	5	E	10
11	5	E	5
12	6	E	10
13	6	E	10
14	6	LE	4
15	6	E	10
16	6	LE	1
18	6	E	12
17	2	LE	3
19	7	E	11
20	7	E	6

### Presentation of results

For all results, “radiologist 1 (R1)” is the individual who interpreted the MRE for the main trial and, “radiologist 2 (R2)” and “radiologist 3 (R3)” refer to radiologists who subsequently re-interpreted the MRE for the current study.

### Small bowel disease presence and extent

Of the 28 newly diagnosed patients, 26 had small bowel disease confirmed by the reference standard (Table 3). Overall agreement with the reference standard for disease presence (averaged between pairs of radiologists) was 68%, (κ = 0.36,) and 43% for disease extent (κ = 0.14,) (Table 3). Of the 45 patients in the relapse cohort, 33 had small bowel disease confirmed by reference standard. Overall agreement with the reference standard for disease presence (averaged between pairs of radiologists) was 78% (κ = 0.56,) and 53% for disease extent (κ = 0.07) (Table 3).

**Table 3.** Per patient Interobserver variability for the presence of small bowel Crohn’s disease against the consensus reference

	Newly diagnosed *N* = 28							Suspected relapse *N* = 45						
	SB Disease presenta *N* = 26				SB Disease absenta *N* = 2			SB Disease presenta *N* = 33				SB Disease absenta *N* = 12		
	R1	R2	R3	% Average Disease Present Agreement (95% CI)b	% Disease Absent Agreement (95% CI)b	% Overall Agreementb	κ	R1	R2	R3	% Average Disease Present Agreement (95% CI)b	% Disease Absent Agreement (95% CI)b	% Overall Agreementb	κ
Small bowel disease presence	22	23	19	69 (50 to 83)	NR	68	0.36	29	25	27	79 (62 to 89)	75 (47 to 91)	78	0.56
Small bowel disease extent	19	17	14	42 (26 to 61)	NR	43	−0.14	24	16	17	45 (30 to 62)	75 (41 to 91)	53	0.07

R1 - Number of reads reported as disease present reads by radiologist 1, R2 – Number of reads reported as disease present by radiologist 2, R3 – Number of reads reported as disease present by radiologist 3.

NR due to small sample size –2 patients without disease (all reads correctly identified no disease in one patient, other patient one read reported false positive disease presence).

a Patient classification by consensus reference standard.

b Average agreement between pairs of radiologist reads with consensus reference standard.

Supplementary Material 1- Appendix 4 shows agreement between the three radiologist readers according to the reference standard classification. Of the 26 (of 28) new diagnosis patients with small bowel disease, three and two radiologists correctly agreed that disease was present in 17/26 (65%) and 4/26 (15%), respectively. In six patients, two radiologists agreed that disease was absent, although they were correct in only 1. In one patient, all three radiologists correctly agreed that disease was absent.

Of the 33 (of 45) relapse patients with small bowel disease, three and two radiologists correctly agreed that disease was present in 24/33 (73%) and 3/33 (9%), respectively. In 11 patients, all three radiologists agreed that disease was absent, which was correct in seven patients and incorrect in 4 ( Supplementary Material 1- Appendix 4).

**Figure 1.**
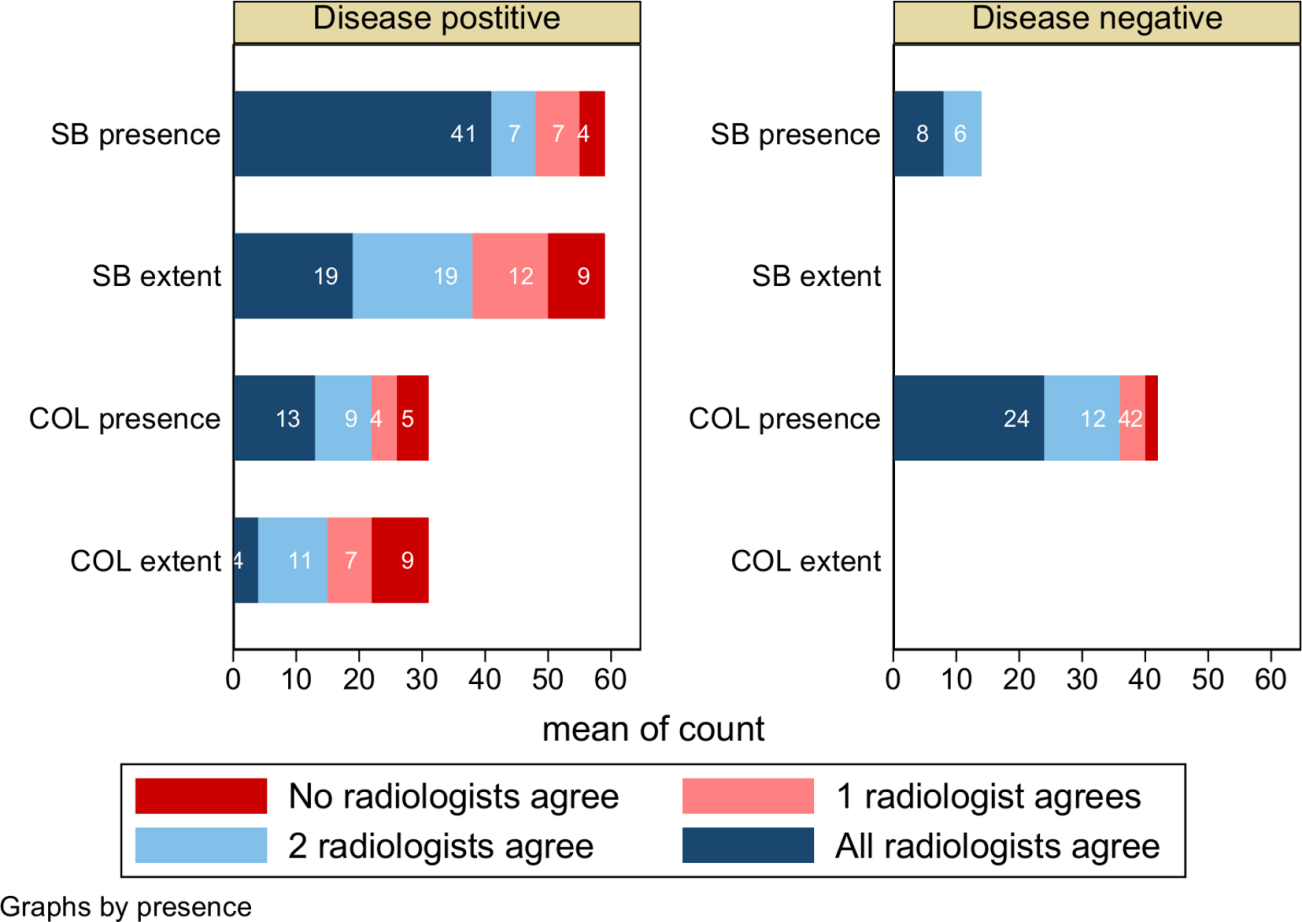
Extent and presence of Crohn’s disease: Agreement between three radiologist reads and the consensus reference standard. Numbers of patients are shown by the consensus reference standard patient classification, where three radiologists (dark blue), two radiologists (light blue), one radiologist (pink) and none of the reads agree (red) with the reference standard. SB (small bowel) COL (colonic).

Combining both cohorts, (Figure 1 Supplementary Material 1- Appendix 5) all three radiologists correctly agreed with the reference standard for disease presence in 41/59 (69%) patients with small bowel disease. However, three-radiologist agreement for disease extent occurred in just 19/59 (32%). All three radiologists correctly agreed with the reference standard in 8/14 (57%) cases of disease absence.

### Segmental small bowel agreement

**Figure 2.**
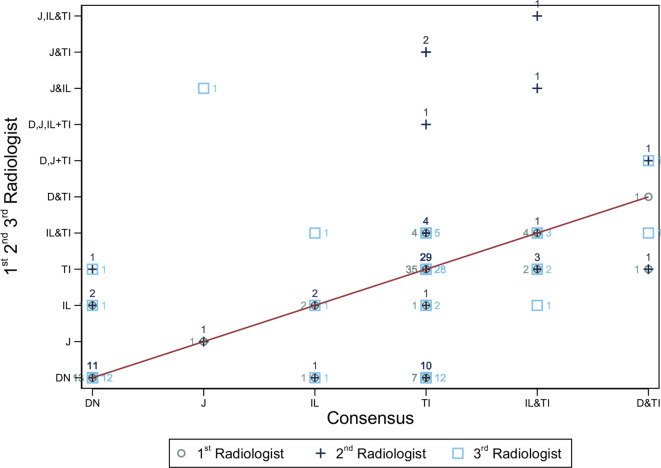
Radiologists and reference standard agreements for small bowel disease extent: Radiologist one read is shown a circle symbol and the number of patients at the disease location. Radiologist two read is shown with a cross-symbol. Radiologist three read is shown with a square symbol. The diagonal line indicates where radiologist reads agree with the consensus on disease location. For example, one patient was found to have disease in the Jejunum (**J**) by the consensus reference. Radiologist 1 and 2 agreed with the reference standard for the patient ( circle and cross-numbered one on diagonal). Radiologist three read reported disease in the jejunum correctly and reported disease in the ileum incorrectly, getting the overall disease extent incorrect for the patient ( box numbered 1). Disease presence: D – Duodenum, J- Jejunum, Il- Ileum, TI- Terminal Ileum, TI– Terminal Ileum, DN- Disease Absent.

Figure 2 demonstrates the segmental disease patterns across all 73 datasets. There were 47 patients with isolated TI disease. Of these, radiologist 1,2 and 3 correctly diagnosed TI disease in 35 (74%), 29 (62%) and 28 (60%) patients respectively. There were 51 instances of single segment disease, correctly diagnosed by radiologist 1, 2 and 3 in 39 (76%), 33 (65%) and 31 (61%) patients, respectively. There were 8 patients with multisegment disease, correctly diagnosed in 5 (63%), 4 (50%) and 5 (63%) by radiologists 1 to 3, respectively.

Supplementary Material 1- Appendix 6 summarizes agreement between radiologists and the consensus findings for individual small bowel segments. There was agreement between all three radiologists and the reference standard in 1/2 (50%), 1/1 (100%), 4/9 (44%) and 36/55 (65%) of patients with duodenal jejunal, ileal and terminal ileal segmental disease, respectively.

### Colonic disease presence and extent

Fourteen newly diagnosed patients had colonic disease confirmed by the reference standard (Table 4). Overall agreement with the reference standard for disease presence (averaged between pairs of radiologists) was 61% (κ = 0.21) and 46% agreement for disease extent (κ = 0.07), 17 patients in the relapse cohort had colonic disease confirmed by consensus reference standard. Overall agreement with the reference standard for disease presence was 60% (κ = 0.20, and 49% agreement for disease extent (κ = 0.02) (Table 4).

**Table 4.** Per patient Interobserver variability for the presence of colonic Crohn’s disease against the consensus reference

	Newly diagnosed *N* = 28							Suspected relapse *N* = 45						
	Colonic Disease presenta *N* = 14				Colonic Disease absenta *N* = 14			Colonic Disease presenta *N* = 17				Colonic Disease absenta *N* = 28		
	R1	R2	R3	% Average disease present agreement (95% CI)b	% Average disease absent agreement (95% CI)b	% Overall Agreementb	κ	R1	R2	R3	% Average disease present agreement (95% CI)b	% Average disease absent agreement (95% CI)b	% Overall Agreementb	κ
Colonic disease presence	8	9	8	43 (21 to 67)	79 (52 to 92)	61	0.21	14	11	11	59 (36 to 78)	61 (42 to 76)	60	0.20
Colonic disease extent	7	5	4	14 (4 to 40)	79 (52 to 92)	46	−0.07	11	7	7	29 (13 to 53)	61 (42 to 76)	49	−0.02

R1 - Number of reads reported as disease present reads by radiologist 1, R2 – Number of reads reported as disease present by radiologist 2, R3 – Number of reads reported as disease present by radiologist 3.

a Patient classification by consensus reference standard.

b Average agreement between pairs of radiologist reads with consensus reference standard.

Supplementary Material 1- Appendix 4 shows agreement between the three radiologist readers according to the reference standard classification. Of the 14 (of 28) new diagnosis patients with colonic disease, three and two radiologists correctly agreed that disease was present in 5/14 (36%) and 4/14 (29%) respectively. In 13 patients, all three radiologists agreed disease was absent, which was correct in 10 patients. Of the 17 (of 45) relapse diagnosis patients with colonic disease, three and two radiologists correctly agreed that disease was present in 8/17 (47%) and 5/17 (29%) respectively. In 12 patients, two radiologists agreed disease was absent, which was correct 10. In 16 patients, all three radiologists agreed disease was absent, which was correct in 14 (Supplementary Material 1- Appendix 4).

Combining both cohorts, all three radiologists correctly agreed with the reference standard for disease presence in 13 out of 31 (41%) patients with colonic disease (Figure 1 Supplementary Material 1- Appendix 7). However, three-radiologist agreement for disease extent occurred in just 4/31 (13%). All three radiologists correctly agreed with the consensus in 24 of 42 patients (57%) with no colonic disease.

### Patient and disease characteristics associated with radiologist agreement for small bowel and colonic disease

#### Small bowel disease presence

Overall, there were 20/73 (27%) patients where two radiologists agreed and 53/73 (73%) where all three radiologists agreed on the presence or otherwise of small bowel disease, independent of the final reference standard classification (Table 5). There were no statistically significant differences in patient characteristics between those in whom three or two radiologists agreed on the presence or absence of small bowel disease. Specifically agreement did not differ according to patient BMI, disease duration or history of previous surgery.

**Table 5.** Association of patient characteristics with radiologist agreement for small bowel disease presence, irrespective of the reference standard

	Small bowel disease presence		Difference in percentage agreement(95% CI)
	Three radiologists agree (*n* = 53)	Two Radiologists agree (*n* = 20)	
**Age - yrs., median (IQR)**	29 (23 to 44)	40 (23 to 49)	−9 (-19 to 10)
**Male n(%)**	29 (55)	11 (55)	0 (-26 to 26)
**BMI –median (IQR)**	22 (19 to 28)	23 (21 to 28)	−1 (-5 to 2)
**Previous enteric surgery n(%)**	23 (43)	7 (35)	8 (-17 to 33)
**Disease duration**>1 year n(%)	34 (64)	10 (50)	14 (-12 to 40)
**Previous disease behaviour** **(Montreal classification)**			
B1 n(%)	29 (55)	15 (75)	−20 (-43 to 3)
B2 n(%)	16 (30)	3 (15)	15 (-5 to 35)
B3 n(%)	8 (15)	2 (10)	5 (-11 to 21)

In 12 of the 73 patients, small bowel disease was reported as absent by all three radiologists (correctly in eight and incorrectly in 4). Of the remaining 61 patients in which at least one radiologist reported small bowel disease, there were 41 (67%) where all three radiologists agreed and 20 (33%) where there was disagreement. Patients with three radiologist agreement for small bowel disease presence were significantly more likely to have multi segment disease, disease measuring more than 5 cm in length, mural thickness>6 mm, stenosis, increased mural T2 signal and peri-mural T2 signal, ulceration, an abnormal mural contrast enhancement pattern comb sign, and increased abnormal mural DWI reported by at least one radiologist (Table 6).

**Table 6.** Association of disease characteristics with radiologist agreement in patients indicated to have small bowel disease by at least one radiologist

	Three radiologists agree small bowel disease present (*N* = 41) 67%	One or two radiologists agree disease present (*N* = 20) 33%	Difference in percentage of disease descriptions Per patient & (95% CI)
Multi segment disease: >1 segment vs ≤1 segment			
Per patient [at least one radiologist reported>1 segment of disease] **a**	49% (20)	0% (0)	49% (34 to 64) p < 0.001
Number of radiologists reporting>1 segment of disease [3,2,1,0]b	[4,4,12,21]	-	
Disease length: ≥5cm compared to <5cm			
Per patient [at least one radiologist reported disease length≥5 cm]a	88% (36)	50% (10)	38% (14 to 62) *p* = 0.001
Number of radiologists reporting disease length≥5 cm [3,2,1,0]b	[29,4,3,5]	[-,4,6,10]	
Wall thickness: ≥6mm compared to <6mm			
Per patient [at least one radiologist reported wall thickness≥6 mm]a	95% (39)	35% (7)	60% (38 to 82) p < 0.001
Number of radiologists reporting wall thickness≥6 mm [3,2,1,0]b	[27,10,2,2]	[-,0,7,13]	
Stenosis			
Per patient [at least one radiologist reported stenosis]a	51% (21)	0% (0)	51% (36 to 66) p < 0.001
Number of radiologists reporting stenosis [3,2,1,0]b	[6,8,7,20]	-	
Perimural T2 signal			
Per patient [at least one radiologist reported abnormal perimural T2 signal]a	25 (61%)	0% (0)	61% (46 to 76) p < 0.001
Number of radiologists reporting abnormal perimural T2 signal [3,2,1,0]b	[7,8,10,16]	-	
Mural T2 signal			
Per patient [at least one radiologist reported abnormal mural T2 signal]a	98% (40)	30% (6)	68% (47 to 89) p < 0.001
Number of radiologists reporting abnormal mural T2 signal [3,2,1,0]b	[21,13,6,1]	[-,3,3,14]	
Ulceration			
Per patient [at least one radiologist reported an ulceration]a	71% (29)	5% (1)	66% (49 to 83) p < 0.001
Number of radiologists reporting an ulceration [3,2,1,0]b	[3,10,16,12]	[-,0,1,19]	
Abnormal contrast enhancement pattern			
Per patient [at least one radiologist reported abnormal contrast enhancement pattern]a	88% (36)	35% (7)	53% (30 to 76) p < 0.001
Number of radiologists reporting abnormal contrast enhancement pattern [3,2,1,0]b	[18,12,6,5]	[-,2,5,13]	
Comb sign			
Per patient [at least one radiologist reported the comb sign]a	68% (28)	10% (2)	58% (39 to 77) p < 0.001
Number of radiologists reporting the comb sign [3,2,1,0]b	[7,10,11,13]	[-,0,2,18]	
**Diffusion signal**			
Per patient [at least one radiologist reported abnormal diffusion signal]a	93% (38)	35% (7)	58% (36 to 80) p < 0.001
Number of radiologists reporting abnormal diffusion signal [3,2,1,0]b	[14,16,8,3]	[0,1,6,13]	

a Number of patients in whom a particular characteristic was noted to be present by at least one radiologist when all 3-radiologists documented small bowel disease was present compared to when only one or two radiologists documented small bowel disease was present.

b Number of radiologists(3, 2, one or 0) who reported the disease characteristic as being present according to whether small bowel disease was documented as being present by all three radiologist compared to documented as being present by one or two radiologist only.

Corresponding data for colonic disease presence is shown in Supplementary Material 1- Appendix 8 & 9.

### Interobserver agreement in length of small bowel and colonic disease

**Figure 3.**
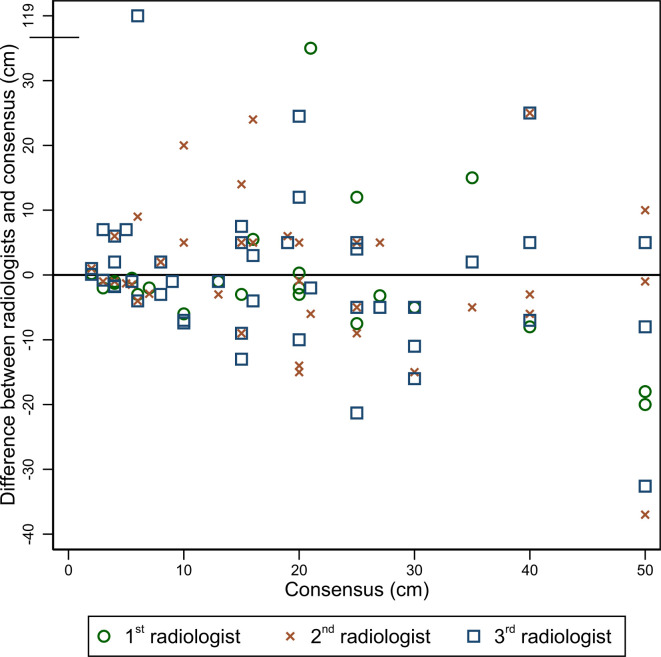
Small bowel disease length: comparison of disease length measurements by three radiologists against the reference standard. Each symbol (first read – circle, second read – cross, third read – square) corresponds to the difference in measured length of abnormal bowel and the reference standard measurement.

**Figure 4.**
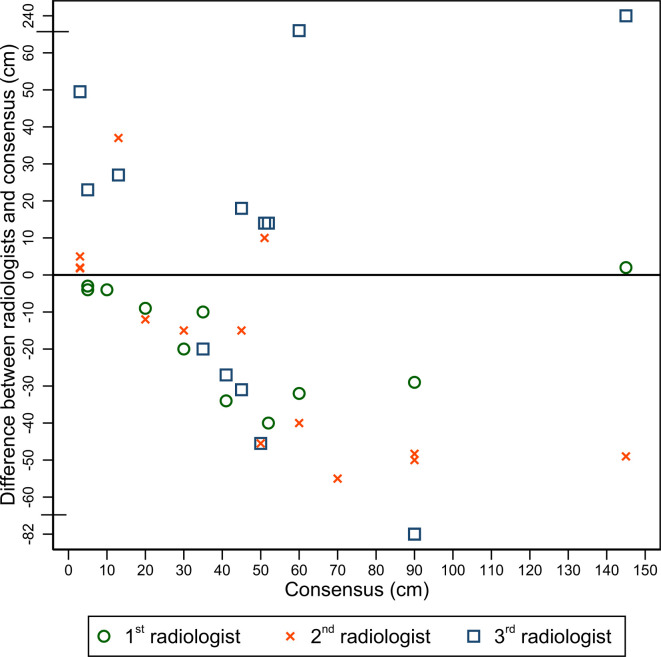
Colonic disease length: comparison of disease length measurements by three radiologists against the reference standard. Each symbol (first read – circle, second read – cross, third read – square) corresponds to the difference of length of abnormal bowel identified in the colon by the radiologist and the consensus reference.

Figures 3 and 4 illustrate variation in measured lengths of small bowel and colonic disease by individual radiologists compared with the consensus reference standard. Radiologists were relatively consistent when measuring the length of disease for small bowel segments below 10 cm in length. Disagreement for length measurement generally increased above 10 cm. Colonic disease length measurements were more variable than small bowel.

### Interobserver agreement for extra-enteric complications

Of the 4 patients with abscesses (by the reference standard), three were identified by all three radiologists and one by two of the radiologists. Of the five patients with fistulae, four patients were identified by all radiologists and one by one radiologist only Supplementary Material 1- Appendix 10).

## Discussion

We investigated agreement between three radiologists’ interpretations and a consensus reference standard for small bowel and colonic Crohn’s disease (CD) presence and extent using 73 prospectively acquired MRE datasets. We found fair and moderate agreement for the presence of small bowel CD in patients with a new diagnosis and those suspected to have luminal relapse respectively. Agreement for colonic disease presence was fair (κ 0.21 for those with a new diagnosis and slight (κ 0.20 for those with suspected luminal relapse. Agreement for disease extent was inferior to that for disease presence.

In a study of 50 MREs and four radiologists, Jensen et al also reported that interobserver agreement for diagnosis of small bowel CD was moderate (κ 0.48).^8^ Schleder et al. (in a study of 84 patients and four radiologists) reported moderate to high interobserver variability for diagnosis of small bowel inflammation (Kendall’s W 0.527–0.823).^9^ and in a study of 33 MREs using four radiologists, Tielbeek et al reported interobserver variability for a variety of disease-specific features such as wall thickness and signal as fair to good (κ 0.30–0.69).^5^ Our results are comparable. Furthermore, our primary analysis compared radiologist agreement with the outcome-based consensus reference standard used in the main trial *i.e.* full agreement could only occur if all three radiologists agreed, and in turn their findings agreed with the final reference standard. The main trial reported a sensitivity of MRE for small bowel disease presence and extent of 97 and 80% respectively.^2^ Our primary analysis therefore incorporates the intrinsic diagnostic accuracy of MRE for Crohn’s disease, and provides a more realistic reflection of clinical utility. We took this approach as high levels of interobserver agreement in the face of low diagnostic accuracy has limited clinical utility.

Our analysis of radiologist agreement independent of the reference standard is interesting and, unsurprisingly, we found better concordance with full radiologist agreement (*i.e.,* concordance between all three radiologists) in 73% (53/73) patients. Once again, the findings are largely comparable to Jensen et al where four observers agreed on the presence or absence of small bowel CD in 27/50 (54%) patients and with Schleder et al where four radiologists agreed on the presence or absence of small bowel CD in 49/84 (58%) patients.^8,9^ Schleder et al also reported interradiologist agreement for small bowel inflammation in the jejunum, ileum and TI as moderate, significant and significant, respectively (Kendall’s W 0.52, 0.72 and 0.82). In the current study, segmental agreement between three radiologists reached 65% for the terminal ileum.

Three radiologist concordance for the presence of colonic disease was improved but remained limited even when the reference standard was ignored; (60% [44/73]) across both new diagnosis and suspected luminal relapse cohorts. MRE has limited utility for diagnosis of colonic disease as technique pivots on luminal contrast timed to optimise small bowel distension. Colonoscopy remains the gold standard for colonic disease.

Confirming our a priori hypothesis, we found that three radiologists were more likely to agree on small bowel disease presence when disease was advanced, *e.g.* multifocal, longer disease length, greater wall thickness and greater mural T2 signal and enhancement. Again, this is perhaps unsurprising. Conversely, earlier disease phenotypes generated higher disagreement, presumably due to failure of a least one radiologist to appreciate subtle disease..^14,15^ The reasons for imperfect agreement between radiologists interpreting are multiple and include dataset quality, interpretation software, radiologist experience, reporting style, disease distribution and severity. Our data suggest radiologist training must include cases of subtle but confirmed disease in order to maximise diagnostic accuracy. A previous study reported that less experienced radiologists (defined as experience of below 700 cases) exhibited greater variability than experienced radiologists for assessment of abnormal lymph nodes, the presence of a comb sign, and appreciation of mural thickness.^16^ Tielbeek et al reported 100 training cases combined with experienced feedback was required to raise inexperienced radiologist sensitivity to acceptable levels.^16^ Radiologists in our main trial met a priori criteria regarding experience and training. We found that radiologist experience (above or below 5 years) did not influence agreement with the reference standard. We also did not find any evidence that patient BMI (with potentially low volumes of intraabdominal fat adversely impacting on enteric evaluation), past surgery or disease duration influenced agreement.

To our knowledge, our study is the first to evaluate interradiologist agreement across various segmental patterns of small bowel disease. Single segment disease was misinterpreted as multisegmental in 8 to 14% of cases dependent on the radiologist, and multisegment disease was misinterpreted as single segment in 37 to 50%. Disease patterns influence treatment. For example, an isolated segment of small bowel disease may be treated surgically whereas multi focal disease is best served by medical therapy. Given the risk of important interobserver variability and the potential for incorrect management, we propose that second opinions and consensus reads should be employed prior to surgical management (most appropriately in the context of multidisciplinary team meetings).

Our study has limitations. While we enlisted a large and geographically wide cohort of radiologists, we compared reads by local radiologists (familiar with local MRE protocols and reporting software) with reads by external radiologists who were less familiar with both. This could arguably influence appreciation of more subtle changes as radiologists will be more comfortable with their local protocols. While κ statistics are used widely to express agreement, they do not always indicate the full clinical implications of findings. We do however report percentage agreement, which will be more intuitive for clinicians. We note that the headline levels of agreement with the reference standard were a little below that of the main METRIC trial.^2^ However, the current study evaluated a smaller portion of the main METRIC cohort and our presented data is based on agreement between multiple radiologists and the reference standard rather than performances of individual radiologist interpretation, as in the main METRIC trial.^11^ Although we analysed patient characteristics associated with identification of small bowel disease by all three radiologists, we did not specifically review cases in which disease was incorrectly missed by all three readers. Such review could give useful insights into the limitations of MRE and how it may be improved and is currently planned. We treated equivocal findings of disease presence/activity as positive findings mirroring the methodology of the main trial. Overall, we had very few equivocal findings (reader 1, 2 and 3 reported equivocal findings in 2, 3 and 7 of the 73 patients for small bowel disease presence). The small bowel was not rated as equivocal for disease presence by more than one reader on any occasion. Therefore the impact of equivocal outcomes on our study was limited.

In conclusion, using data from a multicenter prospective cohort trial, we have demonstrated moderate agreement between radiologists for detecting small bowel disease presence using MRE in newly diagnosed Crohn’s disease patients, and patients with suspected relapse. We demonstrate that variability may impact on the classification of single versus multisegment disease which has implications for optimal patient management. Our data suggests surgical decision making may benefit from a second opinion or consensus report as typically occurs in a multidisciplinary team meeting. We highlight higher variability for evaluation of more subtle disease, which has training implications.

## Supplementary Material

bjr.20210995.suppl-01
