# Supplementary material for: Interobserver variation in the interpretation of magnetic resonance enterography in Crohn’s disease
Source: Br J Radiol. 2022 May 12;95(1134):20210995. doi: 10.1259/bjr.20210995 (PMC12187211; doi:10.1259/bjr.20210995)
Supplement: bjr.20210995.suppl-01 [file bjr.20210995.suppl-01.docx]

Appendix 1: MRE protocol outlining the minimum and optional MRI sequences

| Minimum | Coronal true FISP |
| --- | --- |
|  | Buscopan-20 mg IV, |
|  | Axial and coronal non Fat Sat HASTE |
|  | Coronal Fat Sat HASTE |
|  | Axial diffusion b values 50 and 600 |
|  | Coronal pre and post gadolinium T1 (60–70 sec) |
| Optional | Axial True FISP |
|  | Axial Fat Sat HASTE |
|  | Axial post gadolinium T1 |
|  | True FIP dynamic Motility |

Appendix 2

MRE CRF and interpretation key

**PATIENT CASE REPORT FORM: MRI INTERPRETATION Inter-observer variability**

Anatomical classification: * small bowel from DJ flexure mainly to the left of a diagonal running from the RUQ to LLQ showing typical feathery fold pattern, ** last 10cm of ileum upstream of IC valve/anastomosis

| Overall disease assessment | | | | | | |
| --- | --- | --- | --- | --- | --- | --- |
|  | **Normal** | | **Equivocal** | | **Abnormal** | |
| Confidence of presence | 1 (disease definitely not present) | 2 (disease probably not present) | 3 (disease possibly not present) | 4 (disease possibly present) | 5 (disease probably present) | 6 (disease definitely present) |
| Any small bowel disease PRESENT?-tick confidence box |  |  |  |  |  |  |
| Confidence of activity | 1 (disease definitely not active) | 2 (disease probably not active) | 3 (disease possibly not active) | 4 (disease possibly active) | 5 (disease probably active) | 6 (disease definitely active) |
| If present (confidence score ≥3) is it ACTIVE? tick confidence box |  |  |  |  |  |  |
| Confidence of presence | 1 (disease definitely not present) | 2 (disease probably not present) | 3 (disease possibly not present) | 4 (disease possibly present) | 5 (disease probably present) | 6 (disease definitely present) |
| Any colonic disease PRESENT? tick confidence box |  |  |  |  |  |  |
| Confidence of activity | 1 (disease definitely not active) | 2 (disease probably not active) | 3 (disease possibly not active) | 4 (disease possibly active) | 5 (disease probably active) | 6 (disease definitely active) |
| If present (confidence score ≥3) is it ACTIVE? tick confidence box |  |  |  |  |  |  |

**PATIENT CASE REPORT FORM: MRI INTERPRETATION**

| **Lymphadenopathy (0-3)** | **□0** | **□1** | **□2** | **□3** |
| --- | --- | --- | --- | --- |
| **Abnormal free fluid (Y/N)** | **□Yes** |  | **□No** |  |
| **Abscess present** | **□Yes** |  | **□No** |  |
| ***If yes please state size & location*** | |  | | |
| **Fistula present (circle all that apply)** | **□Yes** |  | **□No** |  |
| ***If yes please circle location*** | Ileo-ileal Ileo-colic entero-cutaneous ileo-vesical  colon-vesical jejuno- jejunal jejuno-colic  Other (state_____________________________) | | | |
| **Extra enteric findings (e.g. aortic aneurysm gallstones, solid organ abnormality, phelgmon)** | **□Yes** |  | **□No** |  |
| ***If yes please state*** |  | | | |

| **PATIENT CASE REPORT FORM: MRI INTERPRETATION**  Please complete for each segment | | | | | | |
| --- | --- | --- | --- | --- | --- | --- |
| **Confidence of disease PRESENCE** | | | | | | |
|  | **Normal** | | **Equivocal** | | **Abnormal** | |
| **Segment** | 1 (disease definitely not present) | 2 (disease probably not present) | 3 (disease possibly not present) | 4 (disease possibly present) | 5 (disease probably present) | 6 (disease definitely present) |
| **Duodenum (Duo)** |  |  |  |  |  |  |
| **Jejunum (J)** |  |  |  |  |  |  |
| **Ileum (I)** |  |  |  |  |  |  |
| **Terminal ileum (TI)*** |  |  |  |  |  |  |
| **Caecum (C)** |  |  |  |  |  |  |
| **Ascending colon (A)** |  |  |  |  |  |  |
| **Transverse colon (T)** |  |  |  |  |  |  |
| **Descending colon (Des)** |  |  |  |  |  |  |
| **Sigmoid (S)** |  |  |  |  |  |  |
| **Rectum (R)** |  |  |  |  |  |  |

*throughout, if TI disease is contiguous for over 10cm count just as TI not TI and ileum

| **PATIENT CASE REPORT FORM: MRI INTERPRETATION**  Please complete for each segment if **confidence scores 3-6 for disease presence** above i.e. present or equivocal | | | | | | |
| --- | --- | --- | --- | --- | --- | --- |
| **Confidence of disease ACTIVITY** | | | | | | |
|  | **Normal** | | **Equivocal** | | **Active** | |
| **Segment** | 1 (disease definitely not active) | 2 (disease probably not active) | 3 (disease possibly not active) | 4 (disease possibly active) | 5 (disease probably active) | 6 (disease definitely active) |
| **Duodenum (Duo)** |  |  |  |  |  |  |
| **Jejunum (J)** |  |  |  |  |  |  |
| **Ileum (I)** |  |  |  |  |  |  |
| **Terminal ileum (TI)** |  |  |  |  |  |  |
| **Caecum (C)** |  |  |  |  |  |  |
| **Ascending colon (A)** |  |  |  |  |  |  |
| **Transverse colon (T)** |  |  |  |  |  |  |
| **Descending colon (Des)** |  |  |  |  |  |  |
| **Sigmoid (S)** |  |  |  |  |  |  |
| **Rectum (R)** |  |  |  |  |  |  |

| PATIENT CASE REPORT FORM: MRI INTERPRETATION Disease Description | | | | | | | | | | | | | | | | | | | | | | | | | | | |
| --- | --- | --- | --- | --- | --- | --- | --- | --- | --- | --- | --- | --- | --- | --- | --- | --- | --- | --- | --- | --- | --- | --- | --- | --- | --- | --- | --- |
| PLEASE COMPLETE FOR EACH DISEASE SITE (DEFINED AS >3CM OF NORMAL BOWEL BETWEEN DISEASE SITES). Please continue for other disease sites if required. USE SCORE DEFINITIONS FROM THE MRI KEY. | | | | | | | | | | | | | | | | | | | | | | | | | | | |
| Location | | **Single Wall thickness (mm) thickest portion** | | **Wall thickening(0-3)** | **Length of abnormal bowel (cm)** | | **Stensois causing functional obstruction (0/1)** | | | **Peri-mural T2 signal**  **(0-3)** | **Mural T2 signal (0-3)** | **Ulceration (0-2)** | | **Contrast enhancement (0-3)** | | **Contrast enhancement pattern (0-3)** | | **Comb sign (0/1)** | | **Diffusion signal (0-3)** | | **Does the segment contain established fibrosis (Y/N)** | | **Segmental disease severity assessment (0-2)** | | **Segment shows active disease (Y/N)** | |
| Duo | |  | |  |  | |  | | |  |  |  | |  | |  | |  | |  | |  | |  | |  | |
| J | |  | |  |  | |  | | |  |  |  | |  | |  | |  | |  | |  | |  | |  | |
| I | |  | |  |  | |  | | |  |  |  | |  | |  | |  | |  | |  | |  | |  | |
| TI | |  | |  |  | |  | | |  |  |  | |  | |  | |  | |  | |  | |  | |  | |
| C | |  | |  |  | |  | | |  |  |  | |  | |  | |  | |  | |  | |  | |  | |
| A | |  | |  |  | |  | | |  |  |  | |  | |  | |  | |  | |  | |  | |  | |
| T | |  | |  |  | |  | | |  |  |  | |  | |  | |  | |  | |  | |  | |  | |
| Des | |  | |  |  | |  | | |  |  |  | |  | |  | |  | |  | |  | |  | |  | |
| S | |  | |  |  | |  | | |  |  |  | |  | |  | |  | |  | |  | |  | |  | |
| R | |  | |  |  | |  | | |  |  |  | |  | |  | |  | |  | |  | |  | |  | |
|  | |  | |  |  | |  | | |  |  |  | |  | |  | |  | |  | |  | |  | |  | |
|  | |  | |  |  | |  | | |  |  |  | |  | |  | |  | |  | |  | |  | |  | |
|  | |  | |  |  | |  | | |  |  |  | |  | |  | |  | |  | |  | |  | |  | |
| PATIENT CASE REPORT FORM: MRI INTERPRETATION Disease Description | | | | | | | | | | | | | | | | | | | | | | | | | | | |
| PLEASE COMPLETE FOR EACH DISEASE SITE (DEFINED AS >3CM OF NORMAL BOWEL BETWEEN DISEASE SITES). Please continue for other disease sites if required. USE SCORE DEFINITIONS FROM THE MRI KEY. | | | | | | | | | | | | | | | | | | | | | | | | | | | |
| Location | **Single Wall thickness (mm) thickest portion** | | **Wall thickening (0-3)** | | | **Length of abnormal bowel (cm)** | | **Stensois causing functional obstruction (0/1)** | **Peri-mural T2 signal**  **(0-3)** | | **Mural T2 signal (0-3)** | | **Ulceration (0-2)** | | **Contrast enhancement (0-3)** | | **Contrast enhancement pattern (0-3)** | | **Comb sign (0/1)** | | **Diffusion signal (0-3)** | | **Does the segment contain established fibrosis (Y/N)** | | **Segmental disease severity assessment (0-2)** | | **Segment shows active disease (Y/N)** |
|  |  | |  | | |  | |  |  | |  | |  | |  | |  | |  | |  | |  | |  | |  |
|  |  | |  | | |  | |  |  | |  | |  | |  | |  | |  | |  | |  | |  | |  |
|  |  | |  | | |  | |  |  | |  | |  | |  | |  | |  | |  | |  | |  | |  |
|  |  | |  | | |  | |  |  | |  | |  | |  | |  | |  | |  | |  | |  | |  |
|  |  | |  | | |  | |  |  | |  | |  | |  | |  | |  | |  | |  | |  | |  |
|  |  | |  | | |  | |  |  | |  | |  | |  | |  | |  | |  | |  | |  | |  |
|  |  | |  | | |  | |  |  | |  | |  | |  | |  | |  | |  | |  | |  | |  |
| Any Additional Comments (if required): | | | | | | | | | | | | | | | | | | | | | | | | | | | |

**MRI Imaging Key**

| **Score** | **0** | **1** | **2** | **3** |
| --- | --- | --- | --- | --- |
| **Mural thickness^a^** | 1-3mm | >3-5mm | >5-7mm | >7mm |
| **Mural T2 signal^b^** | Equivalent to normal bowel wall | Minor increase in signal – bowel wall appears dark grey on fat saturated images | Moderate increase in signal – bowel wall appears light grey on fat saturated images | Marked increase in signal – bowel wall contains areas of white high signal approaching that of luminal content |
| **Perimural T2 signal** | Equivalent to normal mesentery | Increase in mesenteric signal but no fluid | Small fluid rim (<2mm) | Large fluid rim (>2mm) |
| **Mural enhancement pattern** | N/A | Homogenous | Mucosal | Layered |
| **Enhancement^b^** | Equivalent to normal bowel wall | Minor enhancement – bowel wall signal greater than normal small bowel but significantly less than nearby vascular structures | Moderate enhancement – bowel wall signal increased but somewhat less than nearby vascular structures | Marked enhancement – Bowel wall signal approaches that of nearby vascular structures |
| **Lymph nodes^d^** | Absent | Cluster less than 1cm | 1 node greater than 1cm | 3 nodes greater than 1cm |
| **Lymph node enhancement^c^** | Less than nearby vascular structure | Equivalent or greater when compared to nearby vascular structures |  |  |
| **Comb sign** | Absent | Present |  |  |

^a^ Measured using electronic calipers, on HASTE sequences, ^b^ Compared to normal small bowel,

^c^ Compared to nearest vessel, ^d^ Lymph nodes should be measured in short axis diameter

| **Diffusion** | |
| --- | --- |
| **Score** |  |
| **0** | Equivalent to normal bowel |
| **1** | minor increase in signal-bowel wall just visible on highest B value image |
| **2** | moderate increase in signal-bowel wall- easy to see on highest B value images |
| **3** | marked increase in signal- bowel wall very easy to see on highest B value images |

| **Ulceration** | |
| --- | --- |
| **Score** |  |
| **0** | None |
| **1** | Superficial-< 50% wall thickness |
| **2** | Deep-≥50% wall thickness |

| **Functional obstruction = Upstream dilatation** | |
| --- | --- |
| **Score** |  |
| **0** | None |
| **1** | Present |

| **Disease activity** |
| --- |
| **at least one of wall thickening/ increased mural signal/increased mesenteric signal / increased (enhancement mucosal or layered) OR ulceration OR abscess** |

| **Segmental disease severity assessment** | |
| --- | --- |
| **Score** |  |
| **0** | None |
| **1** | Early-superficial ulceration and/or mild wall thickening/ mild increased vascularity |
| **2** | Advanced-transmural disease and/or fistulation and/or stricturing and/or cobblestoning/ and or wall oedema or significant thickening |

Appendix 3:

Reference standard for disease presence in the METRIC trial

Patients were followed for a period of 6 months following recruitment. Each recruitment site then held a series of consensus panels which considered all information collected over the follow-up period including all imaging, endoscopies, surgical findings, histopathology, biochemical markers, and clinical course. The panels included at least one local gastroenterologist, one local radiologist and one radiologist from another recruitment site. The panel recorded whether small bowel or colonic Crohn’s disease was present.

Appendix 4: Radiologist agreement for disease presence with reference to the consensus reference standard classification and patient cohort.

|  | New diagnosis (DP; DN) Total 28 cases | Relapse (DP; DN)  Total 45 cases |
| --- | --- | --- |
| **Small bowel** | 28 (26,2) | 45 (33;12) |
| Three radiologists agree disease present | 17 (17;0) | 24(24;0) |
| Three radiologists agree disease not present | 1 (0;1) | 11 (4;7) |
| Two radiologists agree disease present | 4 (4;0) | 3(3;0) |
| Two radiologists agree disease not present | 6 (5,1) | 7 (2;5) |
|  |  |  |
| **Colon** | 28 (14;14) | 45 (17;28) |
| Three radiologists agree disease present | 6 (5;1) | 9 (8;1) |
| Three radiologists agree disease not present | 13 (3,10) | 16 (2;14) |
| Two radiologists agree disease present | 5 (4;1) | 8 (5;3) |
| Two radiologists agree disease not present | 4 (2;2) | 12 (2;10) |

DP – disease present, DN – disease absent by the reference standard.

Appendix 5: Radiologist agreement for small bowel disease presence against the consensus reference standard classification, both patient cohorts combined

|  | Small bowel Crohn’s disease | | | |
| --- | --- | --- | --- | --- |
|  | Disease present  N=59 | | Disease absent N=14 | All patients N=73 |
| Consensus agreement | Correct  disease extent (N, %) | Correct disease  Present (N, %) | Correct disease absent (N, %) | Overall small bowel disease (present/absent) (N, %) |
| 3 radiologists agree | 19 (32%) | 41 (69 %) | 8 (57%) | 49 (67%) |
| 2 radiologists agree | 19 (32%) | 7 (12%) | 6 (43%) | 13 (18%) |
| 1 radiologists agree | 12 (20%) | 7 (12%) | 0 (0%) | 7 (10%) |
| 0 radiologists agree | 9 (15%) | 4 (7%) | 0 (0%) | 4 (5%) |

Appendix 6: Radiologist agreement for segmental small bowel Crohn’s disease presence against the consensus reference standard, independent of overall disease extent

|  | Small bowel Crohn’s disease present N=59 | | | |
| --- | --- | --- | --- | --- |
| Consensus agreement | Duodenum  N=2 | Jejunum  N=1 | Ileum  N=9 | T. Ileum  N=55 |
| 3 radiologists agree | 1 | 1 | 4 | 36 |
| 2 radiologists agree | 0 | 0 | 2 | 6 |
| 1 radiologists agree | 0 | 0 | 1 | 9 |
| 0 radiologists agree | 1 | 0 | 2 | 4 |

Appendix 7 : Number of radiologist agreement of colonic Crohn’s disease presence with consensus reference standard, both patient cohorts combined

|  | Colonic Crohn’s disease | | | |
| --- | --- | --- | --- | --- |
|  | Disease present patients  N=31 | | Disease absent N=42 | All patients N=73 |
| Consensus agreement | Correct  disease extent (N, %) | Correct disease  Present (N, %) | Correct disease absent (N, %) | Overall small bowel disease (present/absent) (N, %) |
| 3 radiologists agree | 4 (13%) | 13 (42%) | 24 (58%) | 37 (51%) |
| 2 radiologists agree | 11 (35%) | 9 (29%) | 12 (29%) | 21 (29%) |
| 1 radiologists agree | 7 (23%) | 4 (13%) | 4 (9%) | 8 (11%) |
| 0 radiologists agree | 9 (29%) | 5 (16%) | 2 (4%) | 7 (9%) |

Appendix 8: Association of patient characteristics with higher radiologist agreement for colonic disease presence

|  | **Colonic disease presence** | |  |
| --- | --- | --- | --- |
|  | **Three radiologists agree (n=44)** | **Two radiologists agree (n=29)** | **Difference in percentage (95% CI)** |
| **Age - yrs., median (IQR)** | 28 (22 to 46) | 39 (25 to 46) | -9 (-18 to 7) |
| **Male** | 23 (52%) | 17 (59%) | -7% (-30 to 16) |
| **BMI – median (IQR)** | 22 (19 to 24) | 26 (21 to 31) | -4 (-8 to 0) |
| **Previous enteric surgery** |  |  |  |
| Yes | 14 (32%) | 16 (55%) | -23% (-46 to 0) |
| **Disease duration** |  |  |  |
| >1 year | 24 (55%) | 20 (69%) | -14% (-36 to 8) |
| **Previous disease behaviour**  **(Montreal classification)** |  |  |  |
| B1 | 25 (57%) | 19 (66%) | -9% (-32 to 14) |
| B2 | 12 (27%) | 7 (24%) | 3% (-17 to 23) |
| B3 | 7 (16%) | 3 (10%) | 6% (-9 to 21) |

* Bootstrapping used to calculate difference in medians

Appendix 9: Association of disease characteristics with higher radiologist agreement for colonic disease presence

|  | **Three radiologists agree colonic disease present (n=15)** | **Two or one radiologists agree colonic disease present (n=29)** | **Difference in percentage of disease descriptions per patient** |
| --- | --- | --- | --- |
|  |  |  |  |
| **Multi segment disease: >1 segment vs ≤1 segment** | | | |
| Per patient [at least one radiologist reported >1 segment of disease] | 93% (14) | 52% (15) | 41% (19 to 63)  p=0.007 |
| Number of radiologists reporting >1 segment of disease [3,2,1,0] | [9,4,1,1] | [-,5,10,14] |  |
|  |  |  |  |
| **Disease length: ≥5cm compared to <5cm** | | | |
| Per patient [at least one radiologist reported disease length ≥5cm] | 100% (15) | 72% (21) | 28% (12 to 44)  p=0.023 |
| Number of radiologists reporting disease length ≥5cm [3,2,1,0] | [11,2,2,0] | [-,8,13,8] |  |
|  |  |  |  |
| **Wall thickness: ≥6mm compared to <6mm** | | | |
| Per patient [at least one radiologist reported wall thickness ≥6mm] | 100% (15) | 69% (20) | 31% (14 to 48)  p=0.016 |
| Number of radiologists reporting wall thickness ≥6mm [3,2,1,0] | [11,3,1,0] | [-,7,13,9] |  |
|  |  |  |  |
| **Stenosis** | | | |
| Per patient [at least one radiologist reported stenosis] | 7% (1) | 7% (2) | 0% (-16 to 16)  p=1.000 |
| Number of radiologists reporting stenosis [3,2,1,0] | [0,0,1,14] | [-,0,2,27] |  |
|  |  |  |  |
| **Perimural T2 signal** | | | |
| Per patient [at least one radiologist reported abnormal perimural T2 signal] | 40% (6) | 21% (6) | 19% (-10 to 48)  p=0.181 |
| Number of radiologists reporting abnormal perimural T2 signal [3,2,1,0] | [0,3,3,9] | [-,2,4,23] |  |
|  |  |  |  |
| **Mural T2 signal** | | | |
| Per patient [at least one radiologist reported abnormal mural T2 signal] | 80% (12) | 48% (14) | 32% (5 to 59)  p=0.041 |
| Number of radiologists reporting abnormal mural T2 signal [3,2,1,0] | [6,4,2,3] | [-,4,10,15] |  |
|  |  |  |  |
| **Ulceration** | | | |
| Per patient [at least one radiologist reported an ulceration] | 27% (4) | 21% (6) | 6% (-21 to 33)  p=0.654 |
| Number of radiologists reporting an ulceration [3,2,1,0] | [0,2,2,11] | [-,0,6,23] |  |
|  |  |  |  |
| **Abnormal contrast enhancement pattern** | | | |
| Per patient [at least one radiologist reported abnormal contrast enhancement pattern] | 80% (12) | 38% (11) | 42% (15 to 69)  p=0.008 |
| Number of radiologists reporting abnormal contrast enhancement pattern [3,2,1,0] | [3,4,5,3] | [-,1,10,18] |  |
|  |  |  |  |
| **Comb sign** | | | |
| Per patient [at least one radiologist reported comb sign] | 67% (10) | 43% (10) | 24 (-6 to 54)  p=0.131 |
| Number of radiologists reporting comb sign  [3,2,1,0] | [2,2,6,5] | [-,1,9,13] |  |
|  |  |  |  |
| **Diffusion signal** | | | |
| Per patient [at least one radiologist reported abnormal diffusion signal] | 93% (14) | 67% (16) | 26% (5 to 47)  p=0.056 |
| Number of radiologists reporting abnormal diffusion signal [3,2,1,0] | [4,4,6,1] | [-,5,11,8] |  |

Appendix 10: Per patient Inter radiologist variability for disease complications against the consensus reference standard

|  | Disease Complications | | | | | | |
| --- | --- | --- | --- | --- | --- | --- | --- |
|  | Complication  reported | | | | Complication  not reported |  |  |
|  | R1 | R2 | R3 | % Average Present  Agree  (95% CI) | % Absent  Agree  (95% CI) | % Overall  Agree | $\kappa$ |
| Abscess presence (n=4) | 4 | 3 | 4 | 75(30 to 95) | 99  (92 to 100) | 97 | 0.95 |
| Fistula presence (=5) | 4 | 4 | 5 | 80(38 to 96) | 88(78 to 94) | 88 | 0.75 |

R1 – radiologist 1, R2 – radiologist 2, R3 radiologist 3
